# Mechanical and medical imaging properties of 3D‐printed materials as tissue equivalent materials

**DOI:** 10.1002/acm2.13495

**Published:** 2021-12-08

**Authors:** Depeng Ma, Ronghui Gao, Minghui Li, Jianfeng Qiu

**Affiliations:** ^1^ Medical Engineering and Technology Center Shandong First Medical University and Shandong Academy of Medical Sciences Taian P. R. China; ^2^ Health Care Department Taishan Sanatorium of Shandong Province Taian P. R. China; ^3^ Medical Science and Technology Innovation Center Shandong First Medical University and Shandong Academy of Medical Sciences Ji'nan P. R. China; ^4^ Qingdao 3E3D Tech. Co. Ltd. Qingdao P. R. China

**Keywords:** 3D printing, elastic modulus, medical imaging, phantom, tissue equivalent materials

## Abstract

Three materials of polylactic acid (PLA), polyamide 12 (PA12), and light curing resin (LCR) were used to construct phantom using 3D printing technology. The mechanical and medical imaging properties of the three materials, such as elastic modulus, density, effective atomic number, X‐ray attenuation coefficient, computed tomography (CT) number, and acoustic properties, were investigated. The results showed that the elastic modulus for PLA was 1.98 × 10^3^ MPa, for PA12 was 848 MPa, for LCR was 1.18×10^3^ MPa, and that of three materials was close to some bones. In the range of 40∼120 kV, the X‐ray attenuation coefficient of three materials decreased with increasing tube voltage. The CT number for PLA, PA12, and LCR was 144, −88, and 312 Hounsfield units at 120 kV tube voltage, respectively. The density and the effective atomic number product (ρ*Z_eff_) were computed from three materials and decreased in the order of LCR, PLA, and PA12. The acoustic properties of materials were also studied. The speeds of sound of three materials were similar with those of some soft tissues.

## 1 INTRODUCTION

Tissue equivalent materials have been widely used in medical research and clinical simulator to mimic the properties of real tissues. For instance, medical imaging researchers utilize tissue equivalent materials to calibrate equipment and develop new imaging methods.^1^, ^2^ In clinical simulators, tissue equivalent materials play important roles as idealized tissue model to train clinical skills of medical worker.^3^, ^4^ In the above mentioned, tissue equivalent materials were generally constructed into the phantom. The manufacturing method of phantom varied with different materials or applications. For instance, biopolymer phantoms such as gelatin,^5^ gellan,^6^ and agarous^7^ were processed by heating, moulding, and cooling. In quality assurance processes of radiotherapy treatment plans, the anthropomorphic phantom was employed. Its process included combination of moulding, grinding, gluing, and assembling. The traditional manufacturing techniques were known as “subtractive manufacturing” because the process involves removing approach. And, those available phantoms represent “standard” persons. It is difficult to achieve customized production. The manufacturing process of these phantoms is high in costs and is limited to accommodate personalized patient's pathological features. In addition, such phantoms consist of homogenous materials do not simulate the inhomogeneity of different tissue such as bone, muscle, and lung.^3^

3D printing, also called additive manufacturing, is a process that produces objects by adding material in layers. This layer‐by‐layer production method provides greater flexibility and creativity in the design process. 3D printing can significantly speeds up the design and prototyping process. Therefore, 3D printing‐related medical devices have been rapidly applied in many medical fields. The surgical simulation system for anatomical disease models is one of the most promising areas for the clinical application of 3D printing technology.^8^, ^9^ The patient‐specific disease model which is manufactured using 3D printing technology can ensure the precise definition of the disease scope and the detailed reflection of the relevant anatomical structure.^10^, ^11^, ^12^, ^13^ Many studies on the clinical application of 3D printing technology have been reported, such as models cranioplasty^14^ and cerebral aneurysm models^15^ in neurosurgery, patient‐specific instruments within diagnosis and treatment,^16^, ^17^, ^18^, ^19^ and nasopharyngeal swabs for diagnosis of COVID‐19.^20^, ^21^, ^22^ For meeting the requirements of clinical application, the mechanical and medical imaging properties of 3D printing materials must be close to those of real tissue. Unfortunately, compared with conventional tissue equivalent materials such as agarose,^23^ PVC,^24^ and gelatin,^25^ the reports for properties of 3D printing materials, especially medical imaging properties, were still relatively rare. In this paper, the mechanical and medical imaging properties of three 3D printed materials of polylactic acid (PLA), polyamide 12 (PA12), and light curing resin (LCR) were systematically tested and analyzed. These results would make us more familiar with 3D printing materials, as well as providing clear guidance in selecting materials suitable as fat, bone, and soft properties. In addition, these data could also be used as a reference tool to guide the multi‐modal imaging of 3D‐printed materials.

## 2 MATERIALS AND METHODS

### 2.1 3D printing materials and phantoms construction

Three kinds of 3D printing materials of PLA, PA12, and LCR, were purchased from Creality, China. All five colors of PLA were 1.75‐mm filament. The five colors were yellow, red, black, fluorescent red, and fluorescent green. PA12 and LCR were white powder and milky liquid, respectively.

### 2.2 Preparation of samples

PLA samples were fabricated using a fused deposition modeling 3D printer (uPrint SE, Stratasys, Israel). Selective laser sintering 3D printer (S360, UnionTech, China) and stereolithography (SLA) 3D printer (HT600S, Hontai, China) were employed to process PA 12 powder and LCR resin, respectively.

### 2.3 METHODS

The 3D printing material samples with 30 × 10 mm^2^ and a length of 30 mm were made for X‐ray attenuation coefficient *(μ*) testing. The method to measure *μ* of the sample was through digital radiography (CXDI‐55G, Canon, Japan) with dosimeter (Solid dose 400, German).^26^ The *μ* was determined using the following equation:

(1) $$I=I_{0}1n^{\mu x}$$

where x is the thickness of the sample, *I*_0_ and *I* are X‐ray beam intensity before and after transmitted through a sample of thickness of x. A CT scan (Lightspeed, GE, USA) was used to measure CT number of 3D printing material samples at the same X‐ray tube voltage. The elastic modulus and density of the samples were measured according to ASTM D638 and D792 at 25 ± 2°C. The speed of sound and acoustic attenuation coefficient of the samples were measured using 5 MHz ultrasound transducer (AFG3102, Tektronix, USA) in water tank following GB/T15261‐2008.

## 3 RESULTS

**FIGURE 1:**
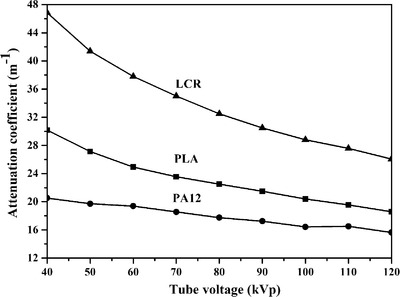
X‐ray attenuation coefficient (*μ*) of three materials at different tube voltage

**FIGURE 2:**
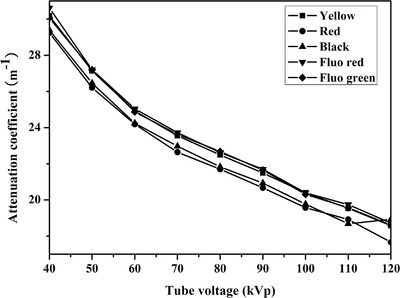
X‐ray attenuation coefficient (*μ*) of polylactic acid (PLA) with different color at different tube voltage

The imaging properties of X‐ray imaging and ultrasound imaging of three 3D printing materials were measured. The X‐ray attenuation coefficient (*μ*) of three materials was tested and calculated for tube voltage from 40–120 kVp at the 100 mA tube current. The *μ* of different materials decreased from 46.8 to 15.6 m^–1^ with increasing tube voltage (Figure 1). At the same time, it could be seen from Figure 1 that the *μ* of LCR was the biggest among three kinds of 3D printing materials, followed by PLA and lastly PA12. The effect of color on *μ* has also been studied in the same material, and results showed that less change of *μ* values was observed when the color of PLA changed (Figure 2).

The CT number is expressed in terms of Hounsfield units (HU) corresponding to the X‐ray attenuation. CT numbers are computed as follows:

(2) $$\mathrm{CT}\;\mathrm{number}=\frac{\mu_{m}-\mu_{w}}{\mu_{w}}\times K$$

where *μ*_m_ is the measured linear attenuation coefficient of the material, *μ*_w_ is the attenuation of water, and *K* (1000) is the scaling factor. Hence, materials that attenuate more than water have positive CT numbers, whereas materials with less attenuation than water have negative CT numbers. CT numbers of materials at 120 kV ranged from −88∼144 HU. CT number for PA12 was −88 HU, PLA was 144 HU, and LCR 312 HU.

The speed of sound and acoustic attenuation coefficient of PLA, LCR, and PA were measured (Table 6). The speed of sound of PLA, about 2246 m/s, is close to that of PA12 (about 2242 m/s) and slightly lower than LCR (about 2427 m/s). However, significantly differences in acoustic attenuation coefficients of above mentioned materials were observed. The acoustic attenuation coefficient for PA12 was 5.44 dB/cm/Hz, LCR was 3.55 dB/cm/Hz, and PLA was 2.31 dB/cm/Hz.

For compound or polymer, their atomic number is called the effective atomic number (*Z*_eff_). The *Z*_eff_ could be estimated by CT measurements using two different X‐ray tube voltages.^27^ Therefore, the *Z*_eff_ of 3D printed materials was computed using the method described by Noblet et al.^28^ Eleven tissue substitute materials of known densities and elemental compositions were scanned with CT to construct the stoichiometric relationship of the function of *ρ* and *Z*_eff_ versus CT number. The curve *ρZ*_eff_ versus CT number led to a monotonic relationship (Equation 3).

(3) $$\begin{array}{ccc} & & \rho Z_{\mathrm{eff}}=5.26\times 10^{-10}H^{3}-1.68\times 10^{-6}H^{2}\\ & & +\;6.44\times 10^{-3}H+7.98 \end{array}$$

where *H* is CT number of material. The CT numbers of three materials were put into Equation 1, and The ρ*Z_eff_ product of the materials was obtained, for LCR 9.85, PLA 8.84, and PA12 7.41.

Compared with traditionally manufactured, the stress‐strain curves of three 3D printed materials exhibited no yield point and were linearly. Elastic modulus is the ratio of stress to strain. As shown in Table 5, elastic modulus of materials ranged from 848∼1980 MPa. The elastic modulus for PA12 was 848 MPa, LCR was 1180 MPa, and PLA 1980 MPa.

## 4 DISCUSSION

### 4.1 X‐ray attenuation coefficient

The *μ* energy dependency curves for material were also plotted against kVp and shown in Figure 1. As shown in Figure 1, the *μ* of all materials decreased with increasing tube voltage. Photoelectric absorption and Compton scattering contribute substantially to X‐ray attenuation in the energy range used in diagnostic radiology when X‐ray transmitted through substance. Moreover, these two interactions decrease with the increase of X‐ray energy.

Another phenomenon observed was *μ*(LCR) > *μ*(PLA) > *μ*(PA12) in the 40–120kVp range at 100 mA. In addition to tube voltage and tube current, the properties of the material (such as effective atomic number *Z*_eff_, density *ρ*) also affect X‐ray attenuation.^29^ In general, substance with higher *Z*_eff_ or *ρ* will absorb more X‐rays, resulting in more X‐ray attenuation. Therefore, we inferred that the *Z*_eff_ and *ρ* of three materials might have order of *Z*(LCR) > *Z*(PLA) > *Z*(PA12), and/or *ρ*(LCR) > *ρ*(PLA) > *ρ*(PA12).

The *μ* change values of PLA with different colors are provided in Figure 2. Less change in the *μ* values was observed when color of PLA changed. Specific material formulations are typically not provided by manufacturers, and, discussing formulations was also outside the scope of this work. However, we could still conclude that the addition of a small amount of pigment would not cause a significant change in *μ* value.

### 4.2 CT number

The CT number is a normalized quantifier of tissue density which is imaged in CT scanner. CT number is obtained by scaling substance *μ* value to more convenient integers and normalizing to *μ* value containing water. Furthermore, CT numbers are displayed as gray‐scale pixels on the digital image. Clinically, the CT number may be of more relevance than *μ* for characterizing the materials.

The CT numbers of PLA, PA12, and LCR were measured and are listed in Table 1. The data in Table 1 showed that the CT number of LCR is the largest, followed by PLA, lastly PA12 among the three materials. The CT numbers of LCR and PLA were positive, indicating that X‐ray attenuation of two materials was stronger than that of water. PA12 had a negative CT number and weaker X‐ray attenuation than water. Table 2 presented CT numbers of some soft and skeletal tissue. The CT number of LCR was 312 HU compared with spongiosa at 262 HU and sternum at 385 HU. PLA and PA12 had value of 144 and −88 HU, comparable with cartilage (102 HU) and adipose tissue (−77 HU), respectively. These suggested that 3D‐printed materials could construct medical phantoms to be equivalent to real tissues.

**TABLE 1:** The CT numbers of polylactic acid (PLA), polyamide (PA), and light curing resin (LCR) at 120 kV and 100 mA

Material	PLA	PA	LCR
CT number (HU)	144	−88	312

Abbreviation: HU, Hounsfield units.

**TABLE 2:** The CT numbers of soft and skeletal tissue^30^

Soft tissue	CT number (HU)	Skeletal tissue	CT number (HU)
Adipose tissue	−77	Red marrow	11
Muscle	40	Cartilage	102
Heart	43	Spongiosa	262
Skin	74	Sternum	385

Abbreviation: HU, Hounsfield units.

### 4.3 Density (*ρ*) and effective atomic number (*Z*_eff_)

Several factors affect X‐ray attenuation. Some are related to the X‐ray beam and the others to properties of the material through which the radiation is passing. The properties of material include the thickness, the density (*ρ*), and the atomic number (*Z*) of material. Table 3 lists the *ρ* and *Z*_eff_ of three materials calculated following Equation 1. As depicted in Table 3, the density descending order of materials was PLA, LCR, PA12; and *Z*_eff_ was the descending order of LCR, PLA, and PA12. The density or atomic number of the material decreases, the attenuation produced by a given thickness decreases. The ρ*Z_eff_ product was also computed from three materials (Table 3) and decreased in the order of LCR, PLA, and PA12. The order of *ρZ*_eff_ agreed with the results obtained from *μ* measurements. The *ρ* and *Z*_eff_ of body tissues from International Commission on Radiation Units and Measurements report 46 are listed in Table 4.^31^ The *ρZ*_eff_ of PLA was close to human muscle, skin, and heart. The *ρZ*_eff_ of PA12 was comparable with red marrow and gastrointestinal (GI) tract. The *ρZ*_eff_ of LCR was similar with cartilage and thyroid.

**TABLE 3:** The *ρ* and *Z*_eff_ of polylactic acid (PLA), polyamide (PA), and light curing resin (LCR)

Material	*ρ* (g/cm^3^)	*Z*_eff_	*ρZ*_eff_
PLA	1.22	7.25	8.84
PA	1.08	6.86	7.41
LCR	1.16	8.49	9.85

**TABLE 4:** The *ρ* and *Z*_eff_ of body tissues

Tissue	*ρ* (g/cm^3^)	*Z*_eff_	*ρZ*_eff_
Red marrow	1.03	7.44	7.66
GI tract	1.03	7.71	7.94
Muscle	1.05	7.85	8.24
Skin	1.09	7.63	8.31
Heart	1.06	7.95	8.43
Cartilage	1.10	8.33	9.16
Thyroid	1.05	9.19	9.65

### 4.4 Mechanical properties

**FIGURE 3:**
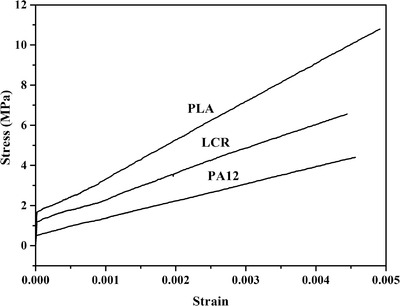
The stress‐strain curves of polylactic acid (PLA), polyamide 12 (PA12), and light curing resin (LCR)

The stress‐strain curves of three 3D‐printed materials are presented in Figure 3. As shown in Figure 3, the stress of three materials increased with the strain linearly. Stress‐strain curves for materials did not exhibit yield point, and the elongation at break was very low (about 0.05%). These effects might be due to layer‐by‐layer production method was prone to separation between layers during the tensile test. To get the elastic modulus, *E*, the measured stress data with strain in the linear elastic region of the material were used. Table 5 lists the elastic modulus of three materials and five types of tissues. PLA and PA12 had the largest and smallest elastic modulus among the three materials, respectively. In addition, all *E* values were seen to be above 100 MPa. Most normal soft tissues (liver, muscle, etc.) have elastic modulus on the order of 10 kPa.^32^ Bone has elastic modulus, more than 10 MPa. Therefore, three 3D‐printed materials were not within the scope of soft tissues. But, it was satisfactory that elastic modulus of three materials was close to bone tissue.

**TABLE 5:** The elastic modulus (E) of materials and tissues

Material	Elastic modulus (MPa)	Tissue	Elastic modulus (MPa)
PLA	1.98 × 10^3^	Muscle	12.8 × 10^−3^
PA	848	Cartilage	12
LCR	1.18 × 10^3^	Meniscus	10–15
―	―	Cancellous Bone	350
―	―	Cortical Bone	17 × 10^3^

### 4.5 Acoustic properties

The acoustic properties (speed of sound, *c*, acoustic attenuation coefficient, *α*) of three samples are shown in Table 6. Three kinds of materials had similar speeds of sound. However, there were obvious differences in acoustic attenuation coefficient of materials. Most tissues in human have speeds of sound around 1500–1600 m/s (Table 7). Compared with human tissues, *c* of samples was close to those of human skin. However, the acoustic attenuation coefficients in samples were between 2.21 and 5.44 dB/cm/Hz, which had a gap with that of real tissue.

**TABLE 6:** The acoustic properties of polylactic acid (PLA), polyamide (PA), and light curing resin (LCR)

Material	Speed of sound, c (m/s)	Acoustic attenuation coefficient, *α* (dB/cm/Hz)
PLA	2246	2.31
PA	2242	5.44
LCR	2427	3.55

**TABLE 7:** The acoustic properties of tissues^33^

Tissue	Speeds of sound, c (m/s)	Acoustic attenuation coefficients, *α* (dB/cm/Hz)
Liver	1590	1.75
Skin	1730	1.99
Muscle	1575	1.68
Bone	4080	7.75
Dentine of teeth	3600	7.92

## 5 CONCLUSIONS

Mechanical and medical imaging properties of three 3D‐printed materials (PLA, PA12, and LCR) were measured. Compared with human tissues, the elastic modulus of the three materials was relatively high (>100 MPa) and could be used to simulate bones. X‐ray attenuation coefficient of materials decreased with increasing tube voltage. And, the order of *μ*(LCR) > *μ*(PLA) > *μ*(PA12) in the 40–120kVp range was observed. The difference of color would not cause a significant change in *μ* value. For CT number, PLA and LCR were comparable with bone tissue when PA12 was close to adipose. The order of the density and the effective atomic number product (ρ* *Z*_eff_) agreed with the results obtained from *μ* measurements. The *ρZ*_eff_ of LCR was similar to that of some bones; PLA and PA12 were close to some soft tissues. The acoustic properties of materials had a gap with that of real tissue. However, speeds of the sound of samples were close to those of human skin. Therefore, 3D printed materials could be used as tissue equivalent materials to simulate some soft and bone tissues, when using 3D printing technology to construct a phantom.

## CONFLICT OF INTEREST

The authors declare that there is no conflict of interest that could be perceived as prejudicing the impartiality of the research reported.

## AUTHOR CONTRIBUTIONS

*Writing the first draft of the manuscript*: Depeng Ma. *Collection and analysis of data*: Depeng Ma, Ronghui Gao, and Minghui Li. *Revising and final approval of the manuscript*: Jianfeng Qiu.

## Supporting information

Supplementary information

## Data Availability

The data that support the findings of this study are available from the corresponding author upon reasonable request.
