# Supplementary material for: Mechanical and medical imaging properties of 3D‐printed materials as tissue equivalent materials
Source: J Appl Clin Med Phys. 2021 Dec 8;23(2):e13495. doi: 10.1002/acm2.13495 (PMC8833282; doi:10.1002/acm2.13495)
Supplement: Supplementary file 1 — Supplementary information [file ACM2-23-e13495-s001.docx]

Mechanical and medical imaging properties of 3D printed materials as tissue equivalent materials

Depeng Ma^1,4 #^, Ronghui Gao^2 #^, Minghui Li^1^, Jianfeng Qiu^1,3,4^

^1^Medical Engineering and Technology Center, Shandong First Medical University & Shandong Academy of Medical Sciences, Taian, 271016, P. R. China

^2^Taishan Sanatorium of Shandong Province, Taian, 271000, P. R. China

^3^Medical Science and Technology Innovation Center, Shandong First Medical University & Shandong Academy of Medical Sciences, Ji’nan, 250118, P. R. China

^4^Qingdao 3E3D Tech. Co., Ltd., Qingdao, 260000, P. R. China

^#^Both authors contributed equally to this work

Author to whom correspondence should be addressed. Jianfeng Qiu

E-mail: [jfqiu100@gmail.com](mailto:jfqiu100@gmail.com)

AUTHOR CONTRIBUTION

Depeng Ma: Writing the first draft of the manuscript.

Depeng Ma, Ronghui Gao and Minghui Li: Collection and analysis of data.

Jianfeng Qiu: Revising and final approval of the manuscript

**ACKNOWLEDGMENTS**

The authors gratefully acknowledged the financial support from the Academic Promotion Programme of Shandong First Medical University (2019QL009), the Qingdao Innovative Talents Projection, the Taishan Scholars Program of Shandong Province (TS201712065) and Science and Technology funding from Jinan (2020GXRC018).
